# Investigation of the Structure–Property Relation of Anthraquinone Dye Molecules with High Dichroism in Guest–Host Liquid Crystal Systems via Computational Methods

**DOI:** 10.3390/ma17246240

**Published:** 2024-12-20

**Authors:** Ruisi Chen, Xintao Guo, Bo Zhang, Ying Liu, Jun Liu

**Affiliations:** 1 AVIC Manufacturing Technology Institute, Beijing 100024, China; chenrs_0225@163.com (R.C.); guoxt001@avic.com (X.G.); zhangbo@avic.com (B.Z.); 2 State Key Laboratory of Organic-Inorganic Composites, Beijing University of Chemical Technology, Beijing 100029, China

**Keywords:** anthraquinone dye, guest–host liquid crystal system, structure–property relation, molecular dynamics simulation, density functional theory

## Abstract

By combining molecular dynamics (MD) simulations and density functional theory (DFT), the influence of dye structure on the optical modulation properties of negative-mode guest–host liquid crystal (GHLC) systems was systematically investigated. Firstly, the reliability of the simulation method was validated by comparing the performance parameters of the GHLC system obtained from simulations with those from experimental results. Subsequently, a series of guest dye molecules, along with their mixtures with negative dielectric anisotropy mesogens, were designed and analyzed. This exploration focused on how variations in dye terminal chain lengths, substitution positions, and substituent group properties affect dye molecular geometry, dye alignment within the host, transition dipole orientation, absorption spectra, and electronic excitation properties. Our findings suggest that dye molecules with a flexible terminal chain substitution of five carbon atoms, positioned at the 2 and 6 locations on the anthraquinone core, exhibit higher order parameters, favorable for enhancing dichroic performance. Moreover, introducing different α-substituents further influences the dye orientation and electronic behavior within the host. These results highlight that structural modifications of anthraquinone-based dyes allow for the design of high-dichroic-ratio materials with customized absorption properties. Overall, our results provide a beneficial understanding of the structure–property relation in GHLC systems, offering valuable guidance for designing high-performance dye molecules and advanced optoelectronic materials in future research.

## 1. Introduction

Incorporating a small amount of dye molecules into a liquid crystal host, forming a GHLC system, imparts the material with distinctive optical and chemical properties [1,2,3]. This technology, compared to other dimming methods such as Polymer Dispersed Liquid Crystal (PDLC) technology [4,5], Suspended Particle Device (SPD) technology [6], and Electrochromic (EC) technology [7], offers several distinct advantages: ultra-fast response times, a wide dimming range, and superior optical performance (low haze and smooth continuous dimming), along with low power consumption and high efficiency [8,9,10]. It has found widespread applications in various fields, including liquid crystal displays [11], optical communications [12], optical modulation [13], and optical sensing [14], and has garnered particular attention in optoelectronics and display technologies [15].

The development of dye-doped liquid crystal systems began in the mid-1960s, with key contributions from George H. Heilmeier, who explored their optical and electro-optical properties [16,17]. These materials feature dichroic dyes that absorb visible light differently along their long and short axes, dissolved within oriented liquid crystal hosts. The alignment of dichroic dyes tends to parallel the liquid crystal molecules (“guest follows host”) [18]. As the orientation of the liquid crystal molecules changes under an electric field, so does the alignment of the dichroic dye molecules. Consequently, the absorption characteristics of the dichroic dye towards incident light vary, enabling effective optical modulation. The light-modulating performance of liquid crystal dyes is influenced by several factors, including the molecular structure of the dye, the intrinsic properties of the liquid crystal mixture, and the solubility of the dye in the liquid crystal host, as well as the equipment and processes used in device fabrication. Among these, the degree of ordered alignment of the dyes within the liquid crystal host is critical to the optoelectronic performance of GHLC systems [19]. Furthermore, suitable dyes must possess a transition dipole moment (TDM) at visible wavelengths, which refers to the vector within the molecular framework where light absorption occurs [20].

The molecular axis of the negative dichroic dye maintains a specific alignment with the electric field of incident polarized light. Maximum absorbance occurs when the electric field of incident polarized light is perpendicular to the main axis of the dye molecule ($A_{⊥}$), and minimum absorbance occurs when the electric field is parallel to the molecular axis ($A_{∥}$). The TDM vector of the dye molecule is orthogonal to the principal molecular axis, exhibiting an $R=A_{∥}/A_{⊥}$ < 1 value in the liquid crystal host [21,22]. Matsui et al. synthesized fluorinated negative dichroic 1,4-bis (amide)anthraquinone dyes, which exhibit this characteristic alignment [23]. For positive dichroic dyes, the TDM of dyes typically aligns parallel to the principal axis of the dye molecule [24,25]. This alignment arises because during the electronic excitation process of the dye molecule, the distribution of the electron cloud in the excited state tends to align with the molecular axis. The maximum absorbance occurs when the electric field of incident polarized light is parallel to the molecular axis ($A_{∥}$), and minimum absorbance occurs when the electric field is perpendicular to the molecular axis ($A_{⊥}$). This type of dichroic structure exhibits a dichroic ratio R > 1, known as positive dichroism. The contrast ratio between the on and off states of the material is determined by the alignment of the TDM vector of the dye within the host liquid crystal system, rather than the orientation of the principal axis of the dye molecule. In practical applications, positive and negative displays are two common display modes. The positive display mode typically consists of positive liquid crystal molecules as the host, which exhibits an absorptive state in the “OFF” state and a transparent state in the “ON” state under an applied electric field. The negative display mode typically consists of negative liquid crystal molecules as the host, which is transparent in the “OFF” state and becomes absorptive in the “ON” state when an electric field is applied. In both modes, the arrangement of liquid crystal molecules and dyes, dielectric anisotropy, and the influence of the applied electric field govern the light absorption and transmission properties. This optical modulation performance plays a crucial role of liquid crystal display technologies. For negative display mode devices, typically composed of negative liquid crystal molecules and positive dichroic dyes, GHLC systems can switch between black and transparent states by incorporating dyes that absorb different wavelengths across the visible spectrum. This system offers high transparency without requiring continuous energy consumption to maintain the display state in the absence of an electric field. When applied in automotive, high-speed rail, aviation, and other transportation sectors, negative dielectric anisotropy mesogens as optical control materials can maintain high transmittance even during power outages or unexpected power loss scenarios, ensuring clear visibility and safety for driving or flight operations. Based on these characteristics, this study primarily focuses on investigating the liquid crystal molecules and dye molecules that operate in the negative display mode.

According to the classification of dye structures, two common types of dye molecules are azo dyes and anthraquinone dyes [26,27]. Azo dyes, distinguished by their rod-like molecular shape, were initially considered by researchers as guest dyes for nematic liquid crystal hosts. Various dye molecules have been designed with different numbers of azobenzene units and various flexible terminal chains to achieve a well-defined rod-like molecular structure while maintaining good compatibility with liquid crystal molecules, which can provide a range of colors [28,29]. However, their practical application is significantly hindered by poor stability, particularly photo-stability [30].

In practical applications, besides considering dye-order parameters, material stability including chemical and photostability is crucial in determining device usage conditions and lifespan. Anthraquinone dyes have been found to be significantly more stable than azo dyes in existing reports, yet despite their ability to align well in liquid crystal molecules, they often exhibit lower dichroism [31]. This phenomenon is due to poor alignment between the TDM of anthraquinone dye molecules and their molecular axes, resulting in a significant angle β [31,32]. Yang et al. [33] successfully synthesized a series of novel dichroic anthraquinone dyes using the conventional Sonogashira reaction, with one yellow dye achieving a dichroic ratio as high as 13.26, the highest reported for anthraquinone dyes to date. With advancements in synthesis, moderate changes in substituents can significantly enhance the alignment of anthraquinone dyes in liquid crystal host [34].

Since the demonstration of the guest–host effect, molecular simulation studies on the alignment of the dye molecules and the mesogenic molecules have seen significant re-search and development [25]. Initially, Wilson et al. [35] employed MD simulations at the all-atom level to study molecular orientation and dipole correlations in the liquid crystal-line mixture E7. Subsequently, Moore et al. [36,37] demonstrated a computational ap-proach combining DFT and MD, offering a potential pathway for the rational design of the dye structure in GHLC systems. Following this, researchers evaluated and optimized the definition of dye principal molecular axes, finding that the surface tensor model provides the best predictive description for molecules of different shapes and flexibility in dyes [38].

Based on existing experimental and simulation work, we acknowledge that there is still a lack of systematic studies on how the molecular structure of anthraquinone dyes influences their order parameter characteristics. To address this gap, this study employs MD simulations combined with DFT to investigate the behavior of a class of dye molecules within liquid crystal hosts. The study describes the geometric structure, spatial orientation, and properties of the dye molecules in their electronically excited states. We systematically examine the effects of factors such as dye terminal chain lengths, substitution positions, and substituent group properties on the dye molecular geometry, alignment within the liquid crystal host, order parameters, transition dipole orientation, absorption spectra, and electronic excitation properties. This exploration aims to uncover the structure–property relationships between dyes and liquid crystal materials. Understanding and quantifying these physical properties are crucial for studying the behavior and applications of dyes in liquid crystal media. Through the quantitative analysis of these factors, we aim to provide valuable theoretical support and guidance for the design of liquid crystal display technologies and other liquid-crystal-based smart materials.

## 2. Materials and Methods

The fully atomistic molecular dynamics (FAMD) study began by randomly distributing liquid crystal host molecules and guest dye molecules in a cubic simulation box using Materials Studio (MS 8.0) software provided by BIOVIA Systems. After energy minimization using the Condensed-phase Optimized Molecular Potentials for Atomistic Simulation Studies (COMPASS) force field, the system was replicated five times to include 200 mesogenic molecules capable of forming a liquid crystal phase, along with five dye molecules in the simulation box. This configuration achieved an effective dye concentration of approximately 3 wt%, which is comparable to that employed in experimental GHLC systems [34]. All molecules were further equilibrated using the COMPASS (Class II) force field in the large-scale atomic/molecular massively parallel simulator (LAMMPS). As depicted in Figure S1, using the D1-C4 system as an example, the snapshots provide a visual representation of the changes in molecular arrangement within the system throughout the equilibration process. The initial configuration of the molecules exhibits a high degree of molecular parallelism, as depicted in Figure S1a. The system was initially equilibrated for 3 ns under the normal volume and temperature (NVT) conditions, as depicted in Figure S1b. The high-temperature equilibration at 400 K helps the system efficiently overcome unrealistic initial structures, such as local energy minima, allowing it to explore the configurational space and approach the low-density state. The elevated temperature increases the kinetic energy of the molecules, enabling them to overcome higher energy barriers and accelerate the relaxation process, promoting faster equilibration. This approach also enhances molecular diffusion and mixing, ensuring uniformity throughout the system and establishing a solid foundation for subsequent simulations as a preprocessing step. The system was then further equilibrated under the normal pressure and temperature (NPT) ensemble, with the pressure set to 0.1 MPa. The system temperature was uniformly reduced from 400 K to 298 K at a rate of approximately 50 K/ns, followed by equilibration under NPT conditions. The Nosé–Hoover thermostat and pressure barostat were used to compress the box to its equilibrium size, as shown in Figure S1c,d. Finally, the system was equilibrated under NVT ensemble for an additional 200 ns to obtain the final equilibrated state for subsequent analysis, as illustrated in Figure S1e. The final density of the system was determined to be ρ≈1.026 g/cm^3^, consistent with experimental results [37]. Van der Waals interactions were calculated using the Lennard–Jones function with a cutoff radius of 9 Å. Newton’s equations of motion were integrated using the Verlet velocity time integration algorithm with a time step of 1 fs. Periodic boundary conditions were applied in all three dimensions to eliminate edge effects during the simulation. The computational analysis data presented below were averaged over the final 40 ns of the NVT equilibration process in the simulation. Further detailed data regarding the molecular dynamics equilibration process can be found in Figure S2.

DFT calculations were performed using the Gaussian 09 software package [39]. Structural optimizations were carried out with the B3LYP functional [40,41,42] and 6-31G (d) basis set, and these optimized geometries were used for subsequent time-dependent density functional theory (TD-DFT) calculations at the same level of theory [43,44]. These calculations were performed on isolated molecules. Molecular orbitals were analyzed using the Multiwfn program (version 3.5) [40]. Under the optimized ground state geometry, the excitation energy and oscillator strength *f* for transitions from the ground state to a range of excited states were calculated. Subsequently, each transition was broadened using a Gaussian function based on these two quantities, and all transitions were overlapped to generate the visible absorption spectrum. The TDM dictates the oscillator strength, which is proportional to the square of the magnitude of the TDM, as illustrated by the following equation:

(1) $$f=\frac{2m_{e}}{3\hbar^{2}}\times \frac{e^{2}}{\varepsilon_{0}}\times \Delta E\times {\left|d\right|}^{2}$$

where *f* represents a dimensionless quantity, the oscillator strength; ΔE represents the energy difference, which is often referred to as the optical gap; $\left|d\right|$ represents the magnitude of the TDM; $m_{e}$ represents the electron mass; ℏ represents the reduced Planck’s constant; e represents the elementary charge; and $\varepsilon_{0}$ represents the vacuum permittivity. Larger transition dipole moment ($\left|d\right|$) results in a stronger absorption. Smaller energy gap (ΔE) (i.e., longer wavelengths) typically leads to weaker absorption. The oscillator strength determines the probability of transitions between excited states. In the absorption process, the stronger the oscillator strength between the ground state and an excited state, the easier it is to absorb electromagnetic waves of the corresponding frequency and transition to that excited state. Therefore, the stronger oscillator strength results in a more pronounced absorption peak in the absorption spectrum. The optical gap of a molecule *E_opt_* refers to the energy of the lowest electronic transition accessible via absorption of a single photon [45,46]. For instance, in typical organic dye molecules, the excitation energy needed for the vertical transition from the S0 to the S1 state, while under the structure of the S0 state minimal point, corresponds to this optical gap. Utilizing the Multiwfn program for hole–electron analysis allows for a graphical representation of the electronic excitation characteristics of molecules, describing the excitation process as “hole → electron” [39,47]. Based on the overlap function *S_r_* between electrons and holes, we quantitatively assess the electronic excitation characteristics. Lu et al. [39,41,42] defined the Sr index, which integrates the *S_r_* function over the entire space. A higher *S_r_* value indicates greater overlap between holes and electrons, while a lower value suggests a more distinct separation. This index ranges from 0 to 1, where 1 signifies perfect overlap and 0 indicates no overlap. The *D* index measures the distance between the centroids of holes and electrons.

The arrangement of dye molecules can be measured using the experimental dichroic ratio (*R*), which is defined as the ratio of absorbances of the dye when aligned parallel (∥) and perpendicular (⊥) to the direction of incident polarized light. These values are employed in Equation (2) to determine the experimental order parameter $S_{exptl}$. An $S_{exptl}$ value of 1 indicates complete alignment of dye molecules, while a value of 0 signifies no alignment of the dye molecules.

(2) $$S_{exptl}=\frac{R-1}{R+2}=\frac{A_{∥}-A_{⊥}}{A_{∥}+2A_{⊥}}$$

In the host mixture, the molecules align along a preferred axis known as the director, which is defined by a unit vector **n** originating from the long axis of the aromatic part of the molecule. For the dye molecules, which exhibit high linearity, we apply the same approach to define the principal axis of the dye molecules. The alignment of the TDM of a dye molecule with respect to the director is schematically shown in Figure S3 [43,44]. The order parameter $S_{\varphi }$ arises from the distribution of angles between the liquid crystal director and the transition dipole moments of the dye molecules. It can be considered as the product of two separate contributions to the order parameter: (i) the alignment of the long axis of the guest dye molecules within the liquid crystal host, and (ii) the alignment of the TDM within the dye molecules, as shown in Equation (3) [20,36,37,38,44,48,49]. In Equation (4), $S_{\theta }$ represents the order parameter describing the alignment of the long axis of the dye molecules with the director of the liquid crystal, where θ is the angle between them. The second Legendre polynomial $P_{2}$ is used, and the brackets 〈..〉 denote an ensemble average over a number of molecules and time. The order parameter $S_{\beta }$ represents the alignment of the TDM of the dye with the long axis of the dye molecule, where β is the angle between them [25,48,49]. The molecules are not static; a dye molecule generally explores a distribution of θ angles that determines the overall molecular alignment, and the TDM of the dye molecule explores a cone around the molecular axis, with the β angle generally being fixed relative to the molecular axis [44]. This is an approximate method for calculating the order parameter $S_{\varphi }$, which assumes that the molecules is either cylindrically symmetric or approximately cylindrically symmetric, with the anisotropy parameter η being zero or close to zero. To verify the applicability and reliability of the system for this method, we analyzed the rotational constants (Table S1), TDM components (Table S2), and the electron density distribution (Figure S4). These results demonstrate that the dye molecules studied exhibit approximate cylindrical symmetry in a statistical sense, and the anisotropy parameter η is close to zero. This supports the applicability of the approximate method to the systems studied in this work. All order parameter values are presented with precision to four decimal places. This approach not only retains finer details of the data but also aligns the results for effective comparison with other experimental and simulation studies.

(3) $$S_{\varphi }=S_{\theta }S_{\beta }=\langle \frac{1}{2}(3\cos^{2}\theta -1)\rangle \left(\frac{1}{2}(3\cos^{2}\beta -1)\right)$$

(4) $$S_{\theta }=\langle P_{2}\left(\cos\theta \right)\rangle =\langle \frac{1}{2}\left(3\cos^{2}\theta -1\right)\rangle$$

## 3. Results

### 3.1. Validation of Simulation Procedure

**Figure 1:**
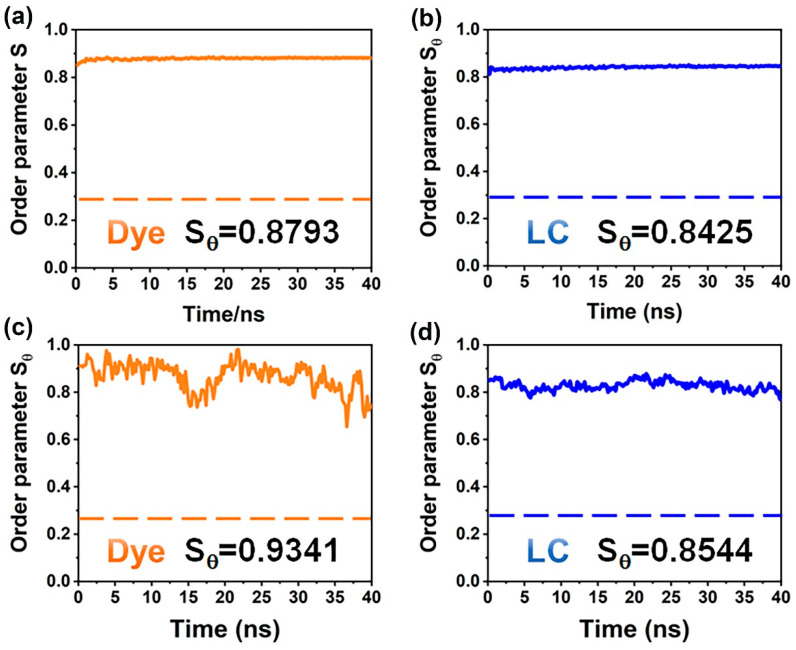
The molecular order parameters of (**a**) the guest dye molecules D–Y and (**b**) the corresponding E7 host molecules; (**c**) the guest dye molecules D4–C4 and (**d**) the corresponding E7 host molecules were calculated and averaged over all 400 E7 host molecules and all 5 dye molecules for each time interval. The insets show the order parameter values obtained by averaging over the last 40 ns of the NVT equilibration process.

To validate the reliability of our simulation approach, we selected a GHLC system, as represented by D-Y system reported by Yang et al., for the verification of our simulation approach. This system utilized a yellow dye molecule D-Y with a high dichroic ratio as the guest and the liquid crystalline mixture E7 as the host [35]. The structure of the D-Y dye molecule is depicted in Figure S5. A summary of the chemical structures and the number of component molecules present in the simulation can be found in Table S3. Using the simulation methods described in the previous section, we calculated the order parameter *S_θ_* for both the E7 mixture and the dye molecules (as shown in Figure 1a,b), as well as the electronic excitation properties of the dye. The simulated results were then compared with the corresponding experimental values for validation. In comparison with the reported results from simulation systems, the order parameter for the E7 host in the D-Y system was measured at 0.8425, which closely aligns with the literature-calculated value of 0.8300, yielding a relative error of 1.51%. Additionally, the order parameter for the dye molecules in the system was found to be 0.8804, indicating a slightly superior spatial arrangement compared to the E7 host, which is consistent with previous research findings [37].

Simultaneously, the optimized DFT structure of the dye was used for TD-DFT calculations to determine the transition dipole moment angles (*β*) with respect to the principal axis of the dye molecule, resulting in a value of 1.62°. Utilizing Equation (3), we calculated the order parameter of the dye molecules to be 0.8793 (as shown in Table S4), which shows a relative error of 9.37% compared to experimental values. This level of accuracy indicates that the simulation method used for the GHLC system provides a reliable description of the order parameter [27].

Through a literature review of the relationship between the synthesized anthraquinone dye structures and their order parameters, we observed that when the terminal alkyl chains of anthraquinone dyes were replaced with alkoxy chains, some dye-order parameters exhibited slight increases [50]. Therefore, we explored the modification of the D-Y dye by substituting the carbon atom adjacent to the aromatic ring in the terminal alkyl chain with an oxygen atom, resulting in the design of dye D4-C4 (as illustrated in Figure S5). The chemical structures and the number of component molecules present in the simulation can be found in Table S3. We then conducted simulations of the order parameters within the E7 host molecules. As shown in Figure 1c,d, the results indicated that the C to O substitution adjacent to the aromatic ring in the terminal chain of anthraquinone dyes increases the nematic order parameter of both the dye molecule and the E7 mixture, with the detailed values presented in Table S5. Consequently, the following research will focus on dye structure design and performance based on the molecular configuration of the dye D4–C4.

### 3.2. Molecular Geometry

**Figure 2:**
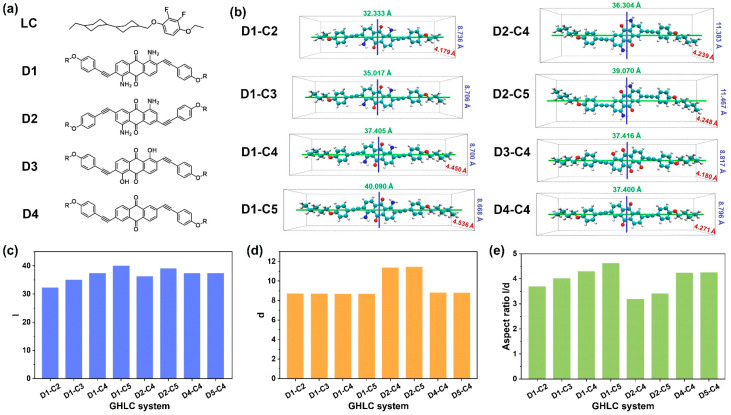
(**a**) The structures and abbreviations of four classes of anthraquinone dye molecules and the mesogenic molecule that form liquid crystal phases in this work. “-R” represents the flexible alkyl chain that is a substituent at the terminal end of the anthraquinone dye molecule. The length of R is determined by the number of carbon atoms in the terminal alkyl chain substituent, ranging from two to five carbon atoms. (**b**) Optimized structures of the dyes showing the van der Waals radii with dashed boxes indicating the molecular lengths and widths defined by the van der Waals surfaces. Green represents the carbon atoms, blue represents the nitrogen atoms, and red represents the oxygen atoms. (**c**–**e**) Bar charts of the molecular lengths, widths, and aspect ratios of the dye molecules.

Figure 2a shows the molecular structure of a mesogenic molecule, labeled as “LC”, alongside four classes of anthraquinone dyes studied in this work. The compound, (R,R)-4-((4-Ethoxy-2,3-difluorophenoxy)methyl)-4′-ethyl-1,1′-bicyclohexane (“LC”), contains a methylene bridge, with two carbon atoms on the benzene ring replaced by fluorine atoms, identifying it as a mesogenic molecule. Under appropriate conditions, this molecule can exhibit a nematic liquid crystalline phase. Due to their large negative dielectric anisotropy, low rotational viscosity, and good solubility, negative liquid crystals are widely used in negative liquid crystal display devices [51]. Typically, simulations of GHLC systems use the positive liquid crystalline mixture E7 as the host [36,48]. In this study, aiming at negative liquid crystal display devices, the host was replaced with mesogenic molecules capable of forming negative liquid crystal phases. This substitution allows for more focused research on the performance of negative-dichroic dye-doped liquid crystal systems. All molecular geometries are the minimum energy structures obtained through DFT calculations. These configurations represent the most stable conformations of molecules, which will be used to analyze their structural properties. In the nomenclature of dye molecules, D1, D2, D3, and D4 respectively represent four classes of anthraquinone dye molecules. The -R groups at both ends of the dyes indicate flexible alkyl chains of varying lengths, with the carbon atom count ranging from two to five. The length of the terminal alkyl chain is denoted as CX, where X represents the number of carbon atoms. In D1 dyes, the amino group substitutes at the 1 and 5 positions, while the 2 and 6 positions are substituted by terminal groups containing alkyne, phenyl, and alkoxy chains of varying lengths. D1–C2, D1–C3, D1–C4, and D1–C5 refer to the first class of anthraquinone dye molecules D1, with terminal chains containing two to five carbon atoms. In D2 dyes, the amino group substitutes at the 1 and 5 positions, while the 3 and 7 positions are substituted by terminal groups containing alkyne, phenyl, and alkoxy chains of varying lengths. D2–C4 and D2–C5 represent the D2 anthraquinone dye molecules with terminal alkyl chains containing four and five carbon atoms, respectively. In D3 dyes, the hydroxyl group replaces the amino group at the 1 and 5 positions, and the 2 and 6 positions are similarly substituted by terminal groups with alkyne, phenyl, and alkoxy chains of different lengths. D3–C4 and D3–C5 denote the D3 anthraquinone dye molecules with terminal alkyl chains containing four and five carbon atoms, respectively. For D4 dyes, the 2 and 6 positions are substituted by terminal groups containing alkyne, phenyl, and alkoxy chains of varying lengths. D4–C4 and D4–C5 correspond to the D4 anthraquinone dye molecules with terminal alkyl chains containing four and five carbon atoms, respectively. Taking dye D1–C2 as an example, the D1–C2 system refers to a GHLC system composed of the dye D1–C2 and the mesogenic molecule “LC”. The same applies to other types of dyes.

The dye molecules exhibit rod-like structures, which can be well described using rectangular dimensions. The optimized structures of dye molecules were visualized using the VMD software [52], as shown graphically in Figure 2b. Molecular lengths were defined as the lengths of the van der Waals surfaces parallel to the minimum moment of inertia (MOI) axes [53], and molecular widths were defined as twice the maximum perpendicular distances from the minimum MOI axes to the van der Waals surfaces. The moments of inertia tensor of the molecules provide the moments of inertia of the three principal axes, where the smallest moment corresponds to the longest axis of the molecule, identified as the principal axis of the dye molecule. The largest eigenvalue corresponds to the shortest axis. In Figure 2b, the colors blue, green, and red denote the three principal axes of the dye molecule, respectively. Molecular lengths, widths, and aspect ratios are calculated as the ratios of these two values, as shown in Figure 2c–e. The detailed values can be found in Table S6.

In the D1 system, as the carbon number of the terminal chain increases from 2 to 5, the molecular width shows minimal change, and the molecular length increases linearly with the corresponding aspect ratio. In the D2 system, due to the change of terminal chain substitution position, the molecular length is slightly reduced compared to the D1 system with the same terminal chain length. Concurrently, the molecular width significantly increases, resulting in a reduced aspect ratio. In the D3-C4 and D4-C4 systems, different substituents are introduced at positions 1 and 5. Compared to D1-C4 dye molecules with the same carbon number of the terminal alkyl chain, minimal changes are observed in both length and width, resulting in minimal impact on the aspect ratio.

### 3.3. Effect of Length of the Terminal Chain of Dyes on the System

**Figure 3:**
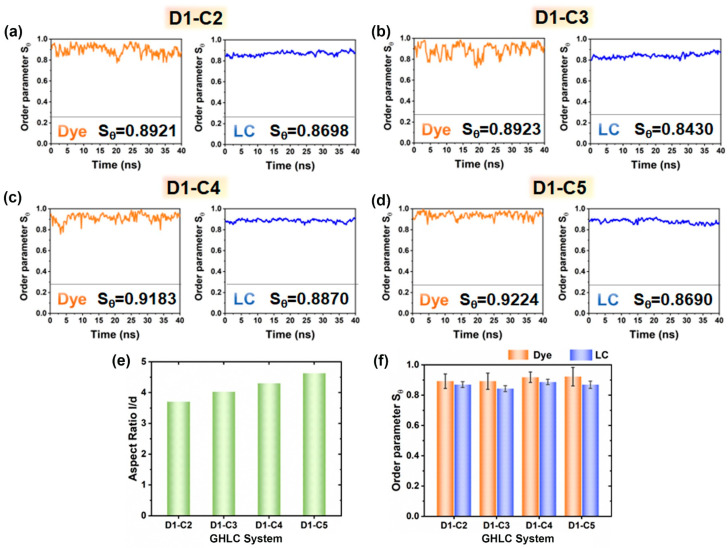
The simulated order parameters of the host molecules “LC” (blue lines) and dye molecules (orange lines) as a function of time for the (**a**) D1–C2 system, (**b**) D1–C3 system, (**c**) D1–C4 system, and (**d**) D1–C5 system. The insets give values obtained by averaging final 40 ns. (**e**) Aspect ratios of the dyes obtained from the van der Waals surfaces of the optimized structures. (**f**) Histogram illustrating the statistical average order parameters for liquid crystal and dye molecules in the D1 systems.

Figure 3a–d present the order parameter values of the dyes and the mesogenic molecules “LC” in systems with the carbon number of the terminal alkyl chain ranging from 2 to 5. As the length of the terminal chain increases, the aspect ratio of the dye molecules also increases, leading to an increase in the order parameter $S_{\theta }$ of both the dye molecules and the mesogenic molecules “LC”, as shown in Figure 3e,f. The flexible terminal alkyl chains facilitate the alignment of dyes within the host. The order parameter of the host molecules in these four systems ranges from 0.8430 to 0.8870, with the order parameter of the dye molecules consistently exceeding that of the host molecules. Furthermore, mesogenic molecules in systems with dyes exhibiting higher order parameters also show higher order parameters.

**Figure 4:**
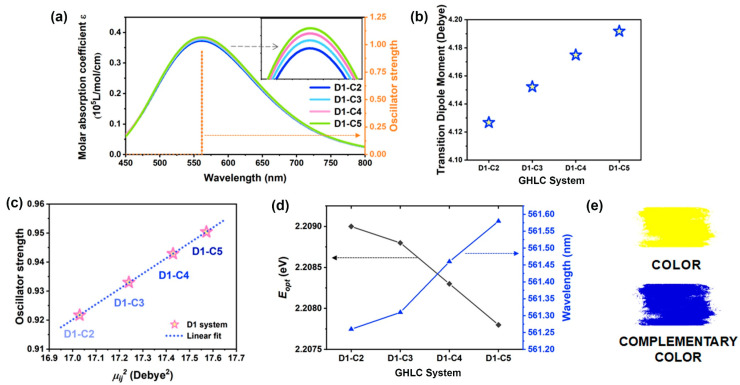
(**a**) Visible absorption spectrum and the oscillator strength corresponding to different terminal chain lengths for D1 systems. (**b**) Modulus of transition dipole moment for different carbon number of terminal alkyl chain in D1 systems (represented by star symbols). (**c**) Oscillator strength as a function of the square of the TDM for D1 systems; the dotted line is a linear fit to the scattered points. (**d**) Optical gap *E_opt_* and corresponding visible absorption peak position of D1 systems. (**e**) Schematic diagram of absorbed color and complementary color of dyes for D1 systems.

To gain a better insight into the electric properties of D1 guest–host systems at the molecular level, DFT calculation was performed. The calculated visible absorption spectra and oscillator strength f of dye molecules with different terminal chain carbon numbers in the D1 systems derived from the TD-DFT calculations are shown in Figure 4a. As the carbon number of the terminal chain increases, the transition dipole moment also increases. Figure 4b illustrates the simulated TDMs for each dye molecule, which increase with the length of the terminal chain. Plotting the square of these TDMs against oscillator strength and fitting them reveals a linear positive correlation between the two variables (Figure 4c). We have simulated the vertical excitation energy from the ground state to the first excited state and determined the corresponding maximum absorption peak position in the visible absorption spectrum, as depicted in Figure 4d. Our findings indicate that as the number of carbon atom in the terminal chain increases, the excitation energy slightly decreases and the absorption peak position undergoes a slight red shift. The absorbed color and the complementary color of the dyes in this type of structure are illustrated schematically in Figure 4e.

**Figure 5:**
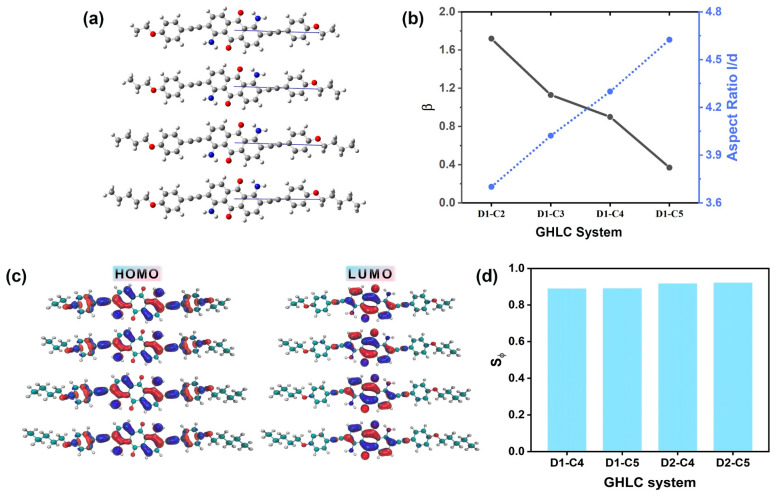
(**a**) Optimized structures of the dyes with the orientations of the TDMs of the visible absorption transitions (blue). From top to bottom, the dyes are D1–C2, D1–C3, D1–C4, and D1–C5. (**b**) Angles, β, between the visible TDMs and the minimum MOI axes of the dyes. (**c**) Optimized structures of the dyes and the orbitals involved in the visible transitions: HOMOs (**left**) and LUMOs (**right**) distributions. Blue represents the positive electron density (positive phase), and red represents the negative electron density (negative phase). From top to bottom, the dyes are D1–C2, D1–C3, D1–C4, and D1–C5. (**d**) The order parameter $S_{\varphi }$ of dyes in four GHLC systems.

Minimum MOI axes were defined as the eigenvectors associated with the minimum eigenvalues of the diagonalized MOI tensors calculated for structures obtained from DFT methods. The orientation of the TDM of the excited dye molecule was determined as illustrated in Figure 5a. Additionally, the angle β between the TDM of the dye molecule and the axis of the minimum MOI of the dye molecule was calculated. A smaller angle indicates that the dye molecule deviates less from its long axis when transitioning from the ground state to the excited state, which corresponds to better alignment with the liquid crystal and an increase in the order parameter. As depicted in Figure 5b, we observed that as the length of carbon number of terminal alkyl chain increases, the aspect ratio of the molecules also increases, with β angles decreasing to 1.72°, 1.13°, 0.9°, and 0.37°. This indicates that the TDMs of the dye molecules in the excited state deviate minimally from the principal axis of the dye molecules, facilitating enhanced dichroic properties. TDDFT calculations reveal that the visible absorption bands of the dye originate from a single HOMO → LUMO transition. These transitions primarily exhibit $\pi \to \pi^{*}$ intramolecular charge transfer properties across all dyes. The orbitals responsible for these transitions are predominantly located on the peripheral anthraquinone ring and its substituents, while the LUMO is centered on the central anthraquinone core and carbonyl groups. These transitions involve similar orbital pairs across all dyes. The visible transitions primarily result from electron density transferring from the substituents towards the anthraquinone core and carbonyl groups. The optimized dye molecular structures and the distribution of the HOMO–LUMO orbitals in the electronic excited state participating in the visible transition are illustrated in Figure 5c. The detailed results, including the associated values $S_{\beta }$ as well as the simulated ordering parameter values $S_{\varphi }$, determined using Equation (3), are all listed in Table S7. When the carbon number of the terminal chain increases from 2 to 5, the order parameter $S_{\varphi }$ of the corresponding dye increases from 0.8909 to 0.9223, as shown in Figure 5d.

### 3.4. Effect of Substitution Position of the Terminal Chain on the System

In addition to controlling the aspect ratio of dye molecules by varying the molecular length of the terminal chain, the substitution positions of substituents on the anthraquinone core also influence the properties of the guest–host system. This section focuses on the impact of different substitution positions on dye molecular properties, spectral performance, alignment of TDMs within the dye molecule, molecular alignment within the GHLC system, and combined results from MD and DFT.

**Figure 6:**
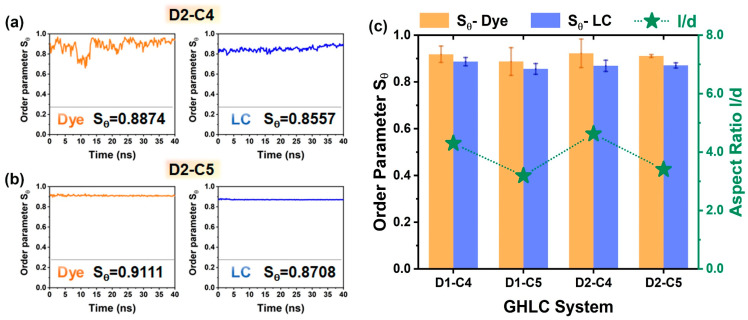
Molecular order parameter of the host and the guest dye simulated versus the host director and averaged over all 200 host molecules and 5 dye molecules of the (**a**) D2–C4 and (**b**) D2–C5 system, respectively. The insets give values obtained by averaging the final 40 ns. (**c**) The effect of the carbon number of the teriminal chain on the order parameter $S_{\theta }$ and the aspect ratio *l*/*d* (represented by the star symbols).

Compared with the D1 system, the substituent position of the terminal chain on the anthraquinone core in the D2 system shifts from position 2,6 to position 3,7, resulting in a slightly different geometric structure of the dye molecules. As the molecular length decreases, the width increases, and the aspect ratio decreases. Comparing the D1 system with the same terminal chain carbon number as the D2 system, the arrangement regularity of substituted anthraquinone dyes at position 3,7 in the liquid crystal body is inferior to that of substituted anthraquinone dyes at positions 2 and 6, as shown in Figure 6a,b. For GHLC systems with the same substitution position, as the carbon number of the terminal chain increases, the aspect ratio of the dye molecules increases. The order parameters of the corresponding dyes in the liquid crystal are positively correlated with the aspect ratio, and their changing trend remain unchanged, as shown in Figure 6c.

**Figure 7:**
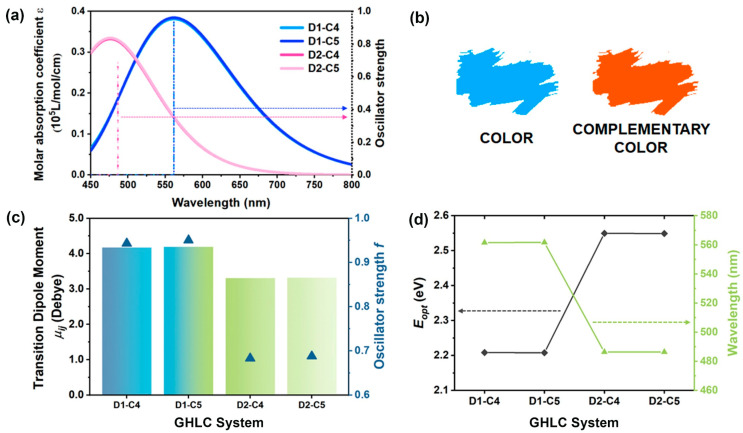
(**a**) Visible absorption spectrum and the oscillator strength corresponding to different terminal chain lengths for D1 and D2 systems. (**b**) Schematic diagram of absorbed color and complementary color of dyes for D2 systems. (**c**) Bar plot of modulus of transition dipole moment and scatter plot of oscillator strength *f* (represented by blue triangles) in D1 and D2 systems. (**d**) Optical gap *E_opt_* and corresponding visible absorption peak position of D1 and D2 systems.

Using the same substituents but varying their positions on the anthraquinone core, we observed the absorption of dyes in the visible light range. Comparing two sets of dyes, D1–C4 with D2–C4 and D1–C5 with D2–C5, it is evident from the molecular structures (Figure 2) that although the molecular lengths are very similar in each group, the geometric shapes differ between them. In the D2 system, due to the change in substitution position, the width of the dye molecules increases, resulting in a reduced aspect ratio. Changes in molecular geometry affect the TDMs of the molecules. As shown in Figure 7c, the TDM of D2 dyes, where substituents are positioned at 3,7, is smaller than that of D1 dyes, and correspondingly, the oscillator strength of D2 is lower than that of D1. Results of optical gaps and maximum absorption peak positions for D1 and D2 systems, as depicted in Figure 7d, reveal that the optical gap of the D2 system is lower, indicating a lower energy difference for transitions to the first excited state compared to the D1 system, resulting in a blue shift in the corresponding maximum absorption peak position. Figure 7a,b display the simulated absorption spectrum for D1 and D2 systems, including their absorption and complementary colors.

**Figure 8:**
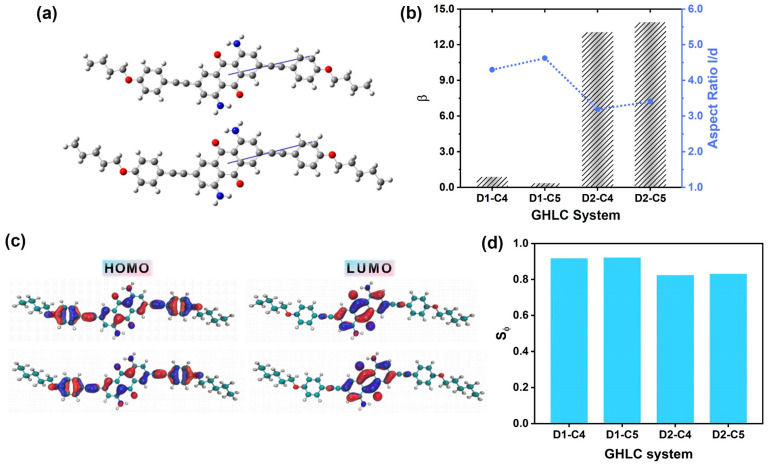
(**a**) Angles β between the visible TDMs and the minimum MOI axes of the dyes. From top to bottom, the dyes are D2–C4 and D2–C5. (**b**) Optimized structures of the dyes with the orientations of the TDMs of the visible absorption transitions (blue). (**c**) Optimized structures of the dyes and the orbitals involved in the visible transitions: HOMO (**left**) and LUMO (**right**) distributions. From top to bottom, the dyes are D2–C4 and D2–C5. Blue represents the positive electron density (positive phase), and red represents the negative electron density (negative phase). (**d**) The order parameter $S_{\varphi }$ of dyes in four GHLC systems.

Changes in molecular geometry not only affect the TDMs of the dyes but also affect the angle β between the TDM and the minimum MOI axis of the dye molecules. The size of the angle β affects how much the dye molecule deviates from its long axis when transitioning from the ground state to the excited state, thereby impacting the ability of the dye to align regularly within a liquid crystal matrix. The angles β for D2–C4 and D2–C5 are 13.08° and 13.91°, respectively, significantly higher than those in the D1 systems, as shown in Figure 8a,b. TDDFT calculations reveal that the visible absorption bands of the dye originate from a single HOMO → LUMO transition. The HOMO orbital is predominantly localized on the external anthraquinone ring and its substituents, while the LUMO is centered on the central anthraquinone core and carbonyl groups, as shown in Figure 8c. The visible transitions primarily result from electron density transferring from the substituents towards the anthraquinone core and carbonyl groups. By combining MD and DFT simulation results using Equation (3), the final ordered parameter values are derived, as illustrated in Figure 8d. It is evident that the position of the substituents significantly affects the order parameters of the dye molecules, particularly impacting the TDMs of the dye molecules, which in turn affects the angle β between the principal axis and the TDM. For dye molecules with substituents at the third and seventh positions of the terminal chain, the degree of molecular alignment is lower compared to the 2,6-disubstituted anthraquinone dye D1. The detailed values can be found in Table S8.

### 3.5. Effect of Substituent Properties on Anthraquinone Dye Cores

**Figure 9:**
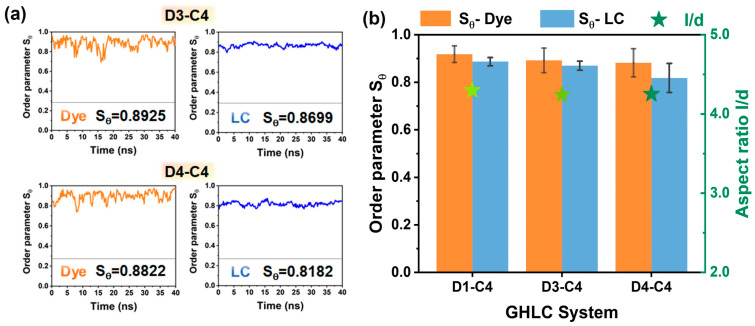
(**a**) Time-dependent evolution of ordered parameters for dye molecules and liquid crystal hosts in D3–C4 and D4–C4 systems with different α-substituents. (**b**) Diagram illustrating the ordered parameters of dyes and liquid crystals in three distinct substituent systems, alongside the corresponding aspect ratios of dye molecules (repre-sented by the star symbols).

Changing substituents on the anthraquinone core alters the aspect ratio of dye molecules and influences their orientation behavior in liquid crystal hosts. By varying α-substituents on the anthraquinone core of dyes, the impact of different substituents on the optoelectronic properties of the GHLC system was studied. In the D1, D3, and D4 systems, the ordered parameters of dyes with α-substituents -NH_2_, -OH, and -H follow the trend -NH_2_ > -OH > -H. The possible reasons for this analysis are as follows. The carbonyl oxygen of dye molecules readily forms hydrogen bonds with amino or hydroxyl groups. Nitrogen atoms have lower electronegativity than oxygen atoms, resulting in weaker attraction to the nucleus towards hydrogen atoms compared to oxygen atoms. Therefore, the hydrogen bonding strength of amino-substituted dye molecules is weaker than that of hydroxyl-substituted ones. When guest dye molecules are introduced into liquid crystal, the number of liquid crystal molecules far exceeds that of dye molecules. Each liquid crystal molecule contains two fluorine atoms, which have high electronegativity and a smaller atomic radius. Compared to the intramolecular hydrogen bonds formed by dye molecules, fluorine atoms compete more effectively with carbonyl oxygen due to their higher electronegativity and stronger hydrogen bonding potential. Consequently, hydrogen atoms on substituents are more likely to form intermolecular hydrogen bonds with fluorine atoms in liquid crystal molecules. In comparison between the two types of substituents, the lower electronegativity of nitrogen atoms compared to oxygen atoms results in hydrogen atoms on α-amino-substituted dye molecules being more prone to forming intermolecular hydrogen bonds with fluorine atoms than those on hydroxyl-substituted ones. Moreover, α-amino-substituted dye molecules have a greater number of hydrogen atoms available for hydrogen bonding compared to hydroxyl-substituted ones, leading to a more orderly arrangement in liquid crystals, as shown in Figure 9a,b. The detailed values of order parameter values for D1–C4, D3–C4 and D4–C4 systems can be found in Table S9.

**Figure 10:**
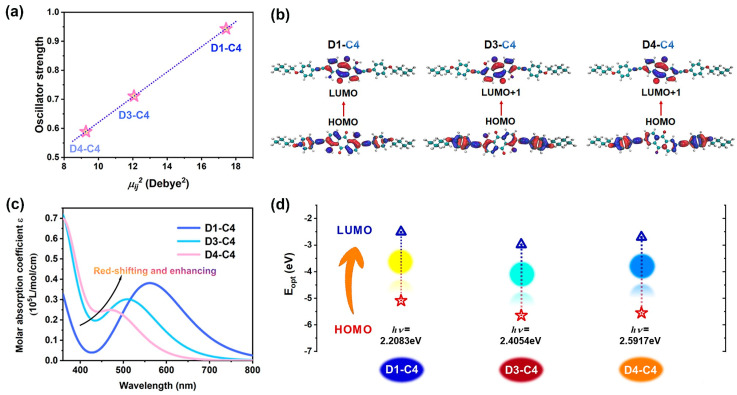
(**a**) Linear fitting graph of oscillator strength *f* versus square of electric TDM. (**b**) Optimized structures of the dyes and the orbitals involved in the visible transitions: HOMO (**left**) and LUMO (**right**) distributions. From top to bottom, the dyes are D1-C4, D3-C4 and D4-C4. (**c**) Visible spectra of different substituent systems. (**d**) Schematic diagram illustrating optical gaps, absorption colors, and complementary colors for different substituent systems.

The formation of conjugated π bonds in dye molecules increases the extent of π-electron delocalization. Adjacent π bonds interact to form large π-conjugated systems, where the energy levels are closely spaced, facilitating easy electron excitation. Consequently, this strengthens the color absorption significantly. In the D1, D3, and D4 systems, the number of conjugated double bonds remains constant, maintaining the conjugation length unchanged. The substituents on the conjugated system can influence the extent of π-electron delocalization, thereby affecting the absorption peak position. Nitrogen and oxygen belong to the second period of elements, and both participate in conjugation through their 2*p* orbitals. Nitrogen has the weakest ability to delocalize lone pair electrons, followed by oxygen. Therefore, the electron-donating effect of substituents follows the order: -NH_2_ > -OH > -H. Electron-donating substituents replacing hydrogen atoms at the α position increase the density of π electron clouds and enhance electron delocalization. This reduces the energy gap between bonding and antibonding orbitals, namely between LUMO and HOMO, resulting in a decrease in the energy required for transitions. As a result, the absorption peak shifts towards longer wavelengths, as shown in Figure 10c. The vertical excitation energies, the optical gaps *E_opt_*, were calculated for the three dye molecules shown in Figure 10d. For D1–C4 and D3–C4 dyes, the optical gap represents the energy difference between the electronic ground state S0 and the first excited state S1. In the case of D4–C4 dye, the first excited state involves a forbidden transition, hence the optical gap corresponds to the energy difference between the electronic ground state S0 and the second excited state S2. The absorption colors and complementary colors of the three dyes are also shown in Figure 10d, with D1, D3, and D4 displaying complementary colors of blue, red, and orange, respectively. The square of the dye TDM is plotted against the oscillator strength *f*, as shown in Figure 10a, revealing a linear positive correlation consistent with the relationship described by Equation (1). Figure 10b displays the contour plots of the HOMO and LUMO orbitals responsible for the visible transitions in the dye. Electrons transfer from the outer anthraquinone ring and its substituents towards the central anthraquinone core and carbonyl groups. According to Table S10, the contributions of the primary transition orbitals in all three systems to the ground state to the lowest excited state transitions exceed 98%, signifying their predominant role. This further confirms that the electronic excitation characteristics can be effectively discussed by focusing on these primary transition orbitals. This similarity also underscores why the distribution of electron–hole pairs aligns closely with the distribution of HOMO–LUMO (or LUMO+1) orbital contour maps.

**Figure 11:**
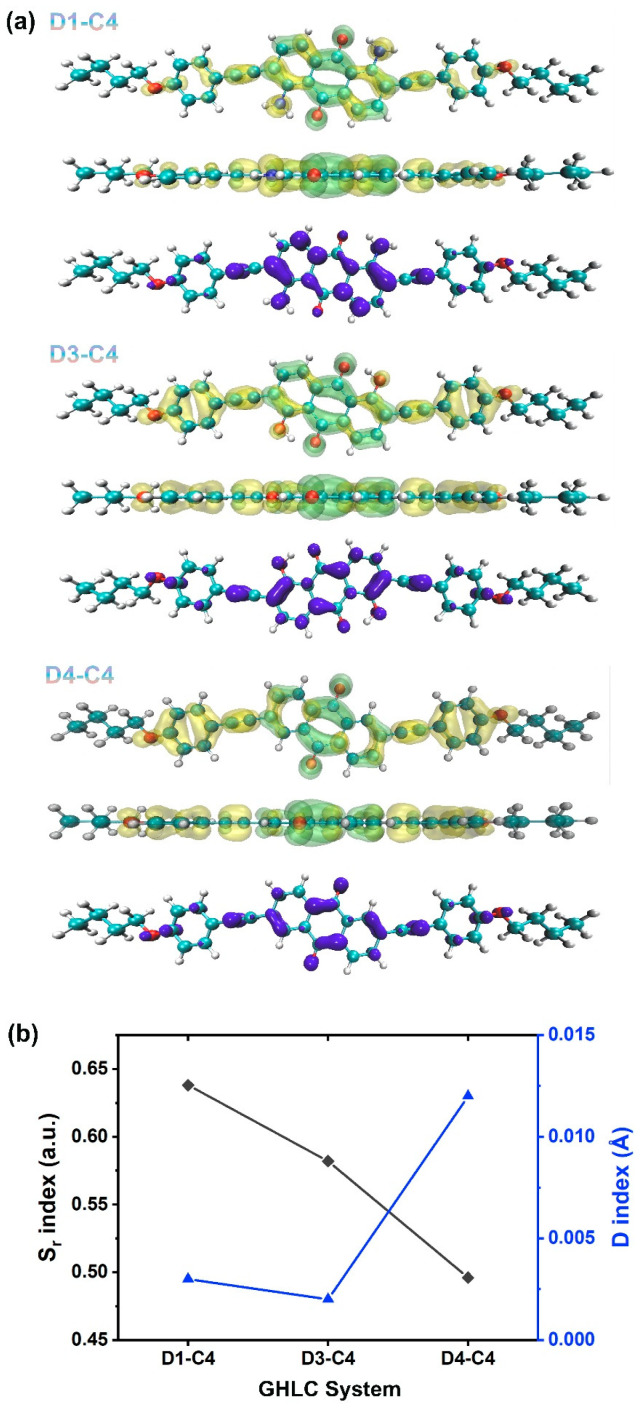
(**a**) The electron–hole analysis of three dyes. For each molecule, the first two images from top to bottom show the frontal and lateral views of the electron–hole contour maps. The yellow area represents the distribution of holes, and the green area represents the distribution of electrons. The third image illustrates the contour map of the overlapping region between electrons and holes (blue area). (**b**) The *S_r_* index and *D* index for the three systems.

We further investigated the electronic excitation characteristics of dye molecules with different substituents, conducting hole–electron analysis to graphically and clearly examine where electrons depart from and where they proceed, as shown in Figure 11a. The distribution of “holes” precisely outlines the departure points of excited electrons, while the distribution of “electrons” accurately depicts their destinations. Figure 11 displays the contour maps of electron–hole diagrams for dyes with different substituents. The transition density contour maps cover the entire system except for the end chains, indicating that the current study of electronic excitation involves a whole transition, with holes and electrons distributed throughout the system. This can be further confirmed by drawing the contour maps of holes and electrons. Based on the overlap function *S_r_* between electrons and holes, the electron–hole overlap contour maps are depicted in Figure 11a. To quantitatively assess the electronic excitation characteristics, we calculated the *S_r_* index and *D* index, as shown in Figure 11b. For dyes D1, D3, and D4, approximately half or more of their electron–hole distributions show nearly perfect overlap, as indicated by their *S_r_* indices ranking from highest to lowest as D1, D3, D4. The *D* indices for all three systems are considerably small, significantly less than the length of a C-C bond. This suggests that there is no significant separation between the distributions of holes and electrons, indicating a local excitation (LE). On the other hand, the high overlap between electrons and holes corresponds to large transition dipole moments (TDMs) and oscillator strengths. This explains why these three systems exhibit significant enhancements in absorption intensity along with a red shift in their absorption spectra peaks (Figure 10c).

## 4. Conclusions

Understanding the structure–property relation of anthraquinone dye molecules in GHLC systems is pivotal. The study employs computational simulation methods to explore key factors within the liquid crystal systems. Through molecular dynamics simulations and quantum chemical calculations, it unveils the orientational characteristics of anthraquinone dye molecules in the liquid crystal phase and their direct correlation with dichroic ratios. Firstly, this research validates the reliability of simulation methods for assessing spatial orientation and electronic properties in GHLC systems. By incorporating dichroic ratio yellow dye molecules into the nematic liquid crystal mixture E7, simulations yield orientation parameters and TDMs closely matching experimental values, thereby confirming the accuracy of our approach in simulating this series of dye molecules. Substituting the carbon atom of the terminal alkyl chain with an oxygen atom enhances molecular alignment, offering potential pathways for optimizing dye structures to enhance performance. Furthermore, the impact of the terminal chain length and the substitution positions on the geometric and electronic properties of dye molecules was investigated. An increased chain length correlates with higher orientation parameters and TDMs, resulting in enhanced dichroic performance and a slight red shift in absorption spectra. Substitution positions significantly influence molecular orientation and optical properties, with dyes substituted at positions 2 and 6 demonstrating higher aspect ratios and order parameters compared to those at positions 3 and 7. Additionally, the study examines the effect of different substituents (-NH_2_, -OH, -H) on the anthraquinone core. These substituents alter the extent of π-electron delocalization, thereby modifying absorption peak positions and optical properties. Among the substituents studied, the amino group exhibits the most significant electron-donating effect, resulting in the highest order parameter and longest wavelength absorption peak. In conclusion, our simulation methods have demonstrated their utility in designing and predicting the performance of GHLC systems. These findings highlight the crucial role of molecular structure in influencing dye optoelectronic behavior, providing an effective framework for the design and optimization of high-performance liquid crystal materials with specific optical properties. We will further refine these methods and improve their accuracy through ongoing comparisons with experimental data.

## Data Availability

The original contributions presented in this study are included in the article/supplementary material. Further inquiries can be directed to the corresponding authors.

## Supplementary Materials

The following supporting information can be downloaded at: https://www.mdpi.com/article/10.3390/ma17246240/s1, Figure S1: Equilibration process of the D1–C4 system. (a) Initial molecular configuration of the system. (b) Initial equilibration under the NVT conditions. (c–d) Further equilibration under the NPT ensemble with pressure set to 0.1 MPa and temperature reduced from 400 K to 298 K. (e) Final equilibration under NVT conditions, maintaining a constant density. Figure S2: Time evolution of the system during the equilibration process: (a) density, (b) temperature, (c) volume, (d) non-bonded interaction energy *E_pair_*, and (e) box length. Figure S3: Schematic representation of the relative orientations of the director n for a nematic liquid crystal system, the long axis of a guest dye molecule (blue), and the TDM of the dye (pink), with angles defined. Figure S4: Electron density distribution in the cross-section perpendicular to the molecular long axis (*x*-axis perpendicular to the plane, yz-plane parallel to the plane). Figure S5: Molecular structures of D-Y for validating simulation method reliability and improved dye structure D4–C4. Table S1: Rotational constants of anthraquinone dye molecules. Table S2: TDM of anthraquinone dye molecules. Table S3: Numbers of component molecules used in the MD simulations with 400 total molecules of the E7 mixture and 5 dye molecules. Table S4: Simulation results of ordered parameter values for E7/D-Y system. Table S5: Simulation results of ordered parameter values for E7/D4–C4 system. Table S6: Lengths and widths of the dyes determined from the van der Waals surfaces parallel and perpendicular to the minimum MOI axes, respectively, along with the resultant aspect ratios. Table S7: Simulation results of ordered parameter values for D1 systems. Table S8: Simulation results of ordered parameter values for D1 and D2 systems. Table S9: Simulation results of ordered parameter values for D1–C4, D3–C4, and D4–C4 systems. Table S10: Analysis of orbital transition contributions in three systems.
