# Supplementary material for: Investigation of the Structure–Property Relation of Anthraquinone Dye Molecules with High Dichroism in Guest–Host Liquid Crystal Systems via Computational Methods
Source: Materials (Basel). 2024 Dec 20;17(24):6240. doi: 10.3390/ma17246240 (PMC11678568; doi:10.3390/ma17246240)
Supplement: Supplementary file 1 [file materials-17-06240-s001.zip › materials-3288529-supplementary.pdf]

Supplementary Information

Investigation of the Structure–Property Relation of Anthraquinone Dye Molecules with High Dichroism in Guest-Host Liquid Crystal Systems via Computational Methods

Ruisi Chen[1], Xintao Guo[1], Bo Zhang[1], Ying Liu,[1] Jun Liu*[2]*

[1]AVIC Manufacturing Technology Institute, Beijing 100024, China

[2]State Key Laboratory of Organic-Inorganic Composites, Beijing University of Chemical Technology, Beijing 100029, China

*E-mail address: liuy535@avic.com; lj200321039@163.com

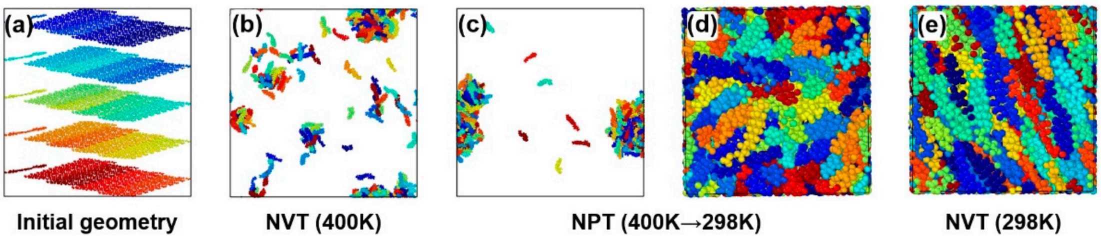

Figure S1. Equilibration process of the D1-C4 system. (a) Initial molecular configuration of the system. (b) Initial equilibration under the NVT conditions. (c-d) Further equilibration under the NPT ensemble with pressure set to 0.1 MPa, and temperature reduced from 400 K to 298 K. (e) Final equilibration under NVT conditions, maintaining a constant density.

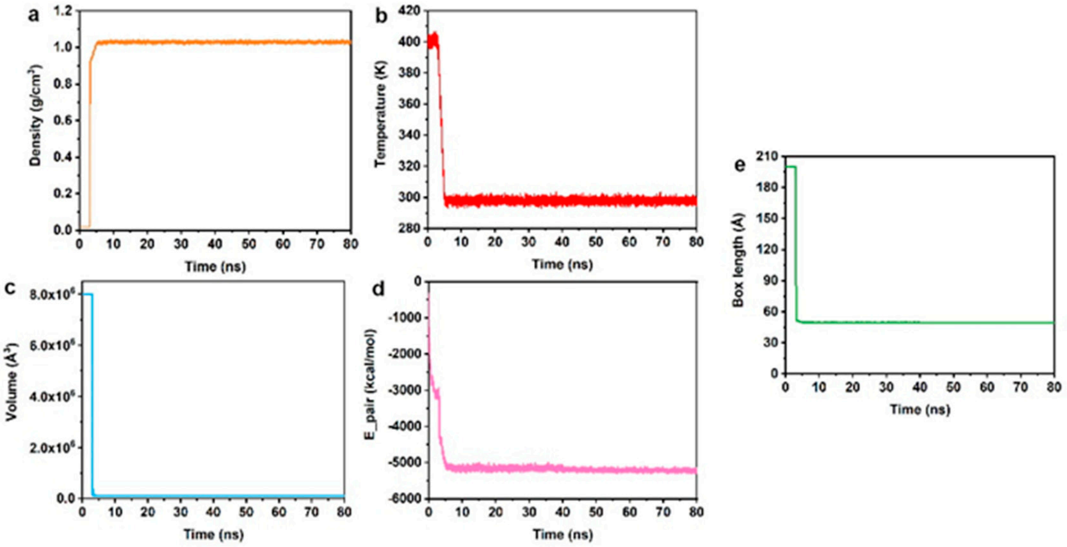

Figure S2 Time evolution of the system during the equilibration process: (a) density, (b) temperature, (c) volume, (d) non-bonded interaction energy E_{pair} and (e) box length.

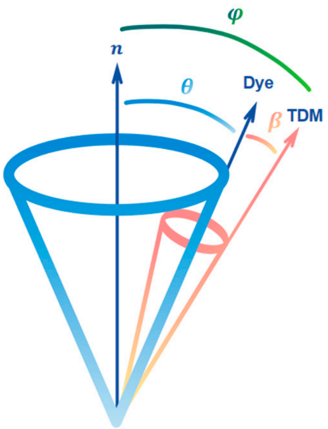

Figure S3. Schematic representation of the relative orientations of the director **n** for a nematic liquid crystal system, the long axis of a guest dye molecule (blue), and the TDM of the dye (pink), with angles defined.

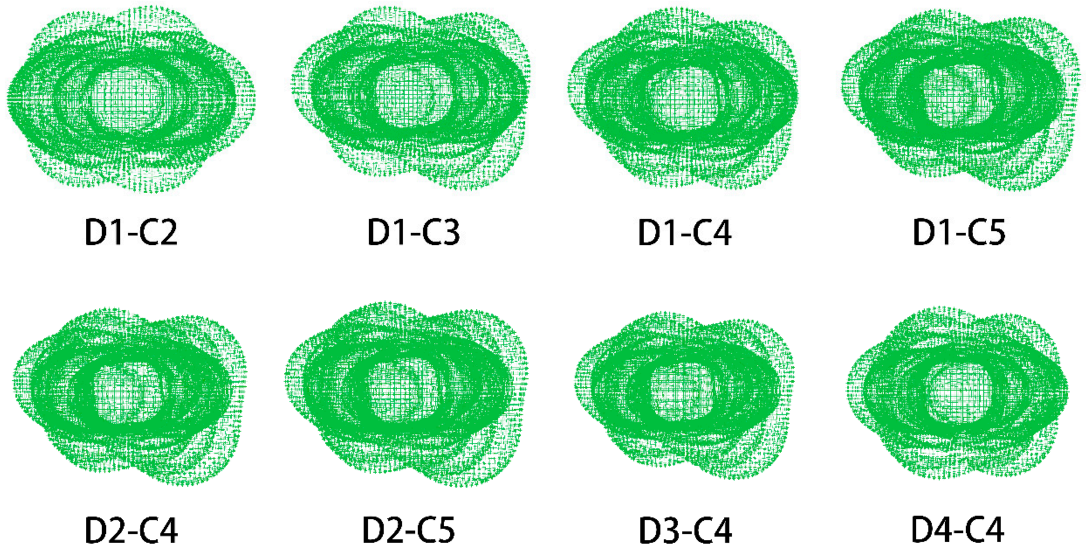

Figure S4 Electron density distribution in the cross-section perpendicular to the molecular long axis. (x-axis perpendicular to the plane, yz-plane parallel to the plane)

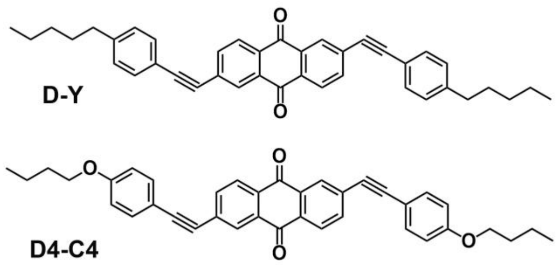

Figure S5. Molecular structures of D-Y for validating simulation method reliability and improved dye structure D4-C4.

Table S1 Rotational constants of anthraquinone dye molecules

Dye	Rotational Constants		
	A	B	C
D1-C2	0.5881	0.0162	0.0158
D1-C3	0.5645	0.0134	0.0131

D1-C4	0.5610	0.0110	0.0108
D1-C5	0.5327	0.0092	0.0091
D2-C4	0.3338	0.0115	0.0111
D2-C5	0.2963	0.0097	0.0094
D3-C4	0.5657	0.0111	0.0109
D4-C4	0.6314	0.0112	0.0110

Table S2 TDM of anthraquinone dye molecules

Dye	TDM		
	$\|TDM\|_x$	$\|TDM\|_y$	$\|TDM\|_z$
D1-C2	3.1250	0.1203	0.0001
D1-C3	5.1515	0.0750	0
D1-C4	5.1743	0.0674	0
D1-C5	5.1912	0.0206	0
D2-C4	4.2248	0.7278	0.0002
D2-C5	4.2168	0.8100	0.0002
D3-C4	3.2635	0.0844	0
D4-C4	4.0256	0.3340	0

Table S3. Numbers of component molecules used in the MD simulations with 400 total molecules of the E7 mixture and 5 dye molucules.

		Before replicate	After replicate
Dye	D-Y	1	5
	D4-C4	1	5
E7 mixture	E7	80	400
E7 mixture component	5CB	44	220
	7CB	20	100
	8OCB	11	55
	5CT	5	25

Table S4. Simulation results of ordered parameter values for E7/D-Y system.

	Physical Quantities	Values (Simulated)
MD	S_θ (Dye)	0.8804
	S_θ (LC)	0.8425
DFT	β	1.62^o
	S_β	0.9988
	S_ϕ (simulated)	0.8793
	S_ϕ (experimental)	0.8040

S_ϕ	Relative error	9.37%

Table S5. Simulation results of ordered parameter values for E7/D4-C4 system.

	Physical Quantities	Values (Simulated)
MD	S_θ (Dye)	0.9342
	S_θ (LC)	0.8544
DFT	β	0.50°
	S_β	0.9999
S_ϕ	S_ϕ (simulated)	0.9341
	S_ϕ (experimental)	/
	Relative error	/

Table S6. Lengths and widths of the dyes determined from the van der Waals surfaces parallel and perpendicular to the minimum MOI axes, respectively, along with the resultant aspect ratios.

GHLC system	Length (Å)	Width (Å)	Aspect ratio l/d
D1-C2	32.333	8.736	3.701
D1-C3	35.017	8.706	4.022
D1-C4	37.405	8.700	4.299
D1-C5	40.090	8.668	4.625
D2-C4	36.304	11.383	3.189
D2-C5	39.070	11.467	3.407
D3-C4	37.416	8.817	4.244
D4-C4	37.400	8.796	4.252

Table S7. Simulation results of ordered parameter values for D1 Systems.

System	l/d	S_θ (Dye)		S_θ (LC)		β/°	S_β	S_ϕ
		Value	S.D.	Value	S.D.			
D1-C2	3.701	0.8921	0.0480	0.8698	0.0190	1.72	0.9986	0.8909
D1-C3	4.022	0.8923	0.0539	0.8430	0.0194	1.13	0.9994	0.8918
D1-C4	4.299	0.9183	0.0350	0.8870	0.0176	0.9	0.9996	0.9179
D1-C5	4.625	0.9224	0.0610	0.8690	030239	0.37	0.9999	0.9223

Table S8. Simulation results of ordered parameter values for D1 and D2 systems.

System	l/d	S_θ(Dye)		S_θ(LC)		β/°	S_β	S_ϕ
		Value	S.D.	Value	S.D.			
D1-C4	4.299	0.9183	0.0350	0.8870	0.0176	0.9	0.9996	0.9179
D1-C5	4.625	0.9224	0.0610	0.8690	030239	0.37	0.9999	0.9223
D2-C4	3.189	0.8874	0.0594	0.8557	0.0231	13.08	0.9295	0.8248
D2-C5	3.407	0.9111	0.0064	0.8708	0.0110	13.91	0.9133	0.8321

Table S9. Simulation results of ordered parameter values for D1-C4, D3-C4 and D4-C4 systems.

	S_θ(Dye)	l	d	l/d	β	S_β	S_ϕ
D4-C4	0.8639	37.400	8.796	4.25193	0.50	0.9999	0.8638
D3-C4	0.8925	37.416	8.817	4.24362	1.07	0.9995	0.8920
D1-C4	0.9183	37.405	8.700	4.29943	0.90	0.9996	0.9179

Table S10. Analysis of orbital transition contributions in three systems.

System	Ground state transition to lowest excited state	Primary transition orbitals	Contribution of primary transition orbitals to the lowest excited state	Lowest excitation energy	Oscillator strength
D1-C4	S0-S1	HOMO-LUMO	99.21%	2.2083	0.9429
D3-C4	S0-S1	HOMO-LUMO+1	99.25%	2.4054	0.7111
D4-C4	S0-S2	HOMO-LUMO+1	98.57%	2.5917	0.5884
